# Advancing okra (*Abelmoschus esculentus* L. Moench) breeding to unlock inherent multi-stress resistance for efficiency and sustainability in agriculture

**DOI:** 10.3389/fpls.2026.1728617

**Published:** 2026-01-29

**Authors:** Phetole Mangena, Abe Shegro Gerrano, Mariette Truter, Lucy Molatudi, Mbali Thembi Gumede, Lindiwe Khoza, Milcah Masemola, Melvin Nyathi, Abueng Moalafi

**Affiliations:** 1 Vegetables, Industrial and Medicinal Plants, Agricultural Research Council, Pretoria, Gauteng, South Africa; 2 Department of Food Security and Safety Focus Area, Faculty of Natural and Agricultural Sciences, North-West University, Mafikeng, South Africa; 3 Department of Plant Production, Soil Sciences, and Agricultural Engineering, School of Science and Agriculture, Faculty of Science and Agriculture, University of Limpopo, Sovenga, Limpopo, South Africa

**Keywords:** abiotic stress, biotic stress, climate change, genetic engineering, genetic variability, polyploidisation, sustainable agriculture

## Abstract

Drought, pests and diseases constitute significant threats to food security, affecting crop growth and development, yield, grain quality, and causing a myriad of adverse physiological and biological effects in okra (*Abelmoschus esculentus* L. Moench) and many other crops. In 2024, the global okra production recorded approximately 11.23 million tons, with India leading the charts, accounting for over 70% of the world production due to environmental constraints. However, significant breeding advancements are being explored in mitigating the effects of biotic and abiotic stresses through the development of stress resilient varieties. Okra breeding for crop diversification face unique challenges characterized by genetic bottlenecks, complex trait inheritances, long breeding cycles and lack of confirmed inherent stress-resistant genes required for multi-stress tolerance. Emerging reports point to progressing breeding through modern techniques like marker-assisted selection (MAS) and genetic modification to develop varieties resistant to drought, pests and diseases. Therefore, this review outlines okra’s potential resistance to living and non-living stress factors, defines genes and mechanisms for possible effective mitigation, and challenges in conferring gene-mediated resistance. We propose efficient breeding strategies further required to enhance growth and productivity of okra, while guaranteeing a sustainably enhanced crop value chain under both favorable and unfavorable climate and environmental conditions.

## 1 Introduction

The biotic and abiotic environmental stress factors remain the most pervasive and destructive phenomena that continue to inflict growth and yield perturbations in agriculture worldwide. These stresses include inorganic factors such as extremely high or low temperatures, drought or flooding, salinity, nutrient deficiency or metal toxicity, as well as bacteria, viruses, fungi, insects and weeds that constitute an array of organic stress factors. However, numerous studies have examined the impact of single or individual stress on crops, than multiple stresses that interact in various ways, causing devastating adverse effects on the plant’s normal metabolism, growth and yield. Iqbal et al. (2021) reported that this plethora of environmental cues pose serious challenges on crops in achieving their full genetic potential for growth and reproduction. One such environmental problem is the recurrent attack by pests, and often with the concomitant impact of drought, on the cultivation and harvesting of crops such as okra (*Abelmoschus esculentus* L. Moench). Okra, commonly known as “Lady’s Finger” belongs to the family Malvaceae which includes several other economically important crops like cotton (*Gossypium hirsutum* L.), cocoa (*Theobroma cacao* L.) and a popular tropical Southeast Asian fruit, durian (*Durio zibethinus*), known for its distinctive strong odor, as well as its unique flavor. Other notable members of this family include kenaf (*Hibiscus cannabinus*) used for fiber production, kola-nut (*Cola nitida*) used for manufacturing of beverages and flavoring agents, and hibiscus plants (*Hibiscus* spp.) utilized for ornamental purposes (Das and Islam, 2019; Basheer et al., 2021). However, many species within the Malvaceae family, with the inclusion of okra, still need to be fully explored as potential medicinal, food and feed crops (Basheer et al., 2021), since they exhibit valuable nutritional, health and ecological values. Current efforts of crop diversification involving okra breeding, nevertheless, face unique challenges characterized by genetic bottlenecks, complex trait inheritance, long breeding cycles, poor agronomic practices, and the lack of confirmed inherent resistance genes required to confer tolerance to multi-stress factors.

The advent of genetic and/or molecular breeding technologies, particularly, genomic selection and gene editing (Lamichhane and Thapa, 2022) have significant potential on improving the generation of mutant lines or genetically improved varieties that are less susceptible to adverse environmental conditions. Traditional breeding techniques like hybridization, back cross breeding, recurrent selection, mass selection and pure-line selections (Mustafa et al., 2021; Prabhu et al., 2023; Anand et al., 2023) are some of the approaches augmented by their integration with these modern plant breeding methodologies. However, we argue in this paper that this integration is not yet fully harnessed for the attainment of improved genetic variability and diversity in okra to generate cultivars showing resistance to unfavorable conditions during large and small-scale cultivations. It is worth noting that approaches such as marker-assisted selection, genetic engineering, mutation breeding and genomic editing, if thoroughly implemented in okra breeding, can allow breeders to identify and transfer specific genes associated with multi-stress tolerance to unlock new opportunities for successful development of new varieties. This much anticipated genetic manipulation, associated with the identification and mapping of various genes and quantitative trait loci (QTL) (Prabhu et al., 2023) with accompanying multiple biotic and abiotic constraints analyses may also lead to the discovery of valuable and abundant growth-yield related DNA marker traits. Therefore, this paper review historical and recent investigations into the consequences of multi-stress factors in okra by focusing on both physiological and biological effects, genetic variations and mechanisms shaping those variations. Furthermore, to share some insights on these issues, we discuss previous, current and future advancements in okra breeding with a particular emphasis on genomic breeding strategies to unlock inherent stress-resistant genes and the impact of multi-stresses that are persistently affecting growth and yield of this crop, particularly, to circumvent them, and improve traits such as higher yield, disease resistance and adaptability to climate change for purposes of realizing its genetic potential under commercial or small-scale agricultural setting.

## 2 Outlook of okra production and benefits for sustainable agriculture

**Figure 1:**
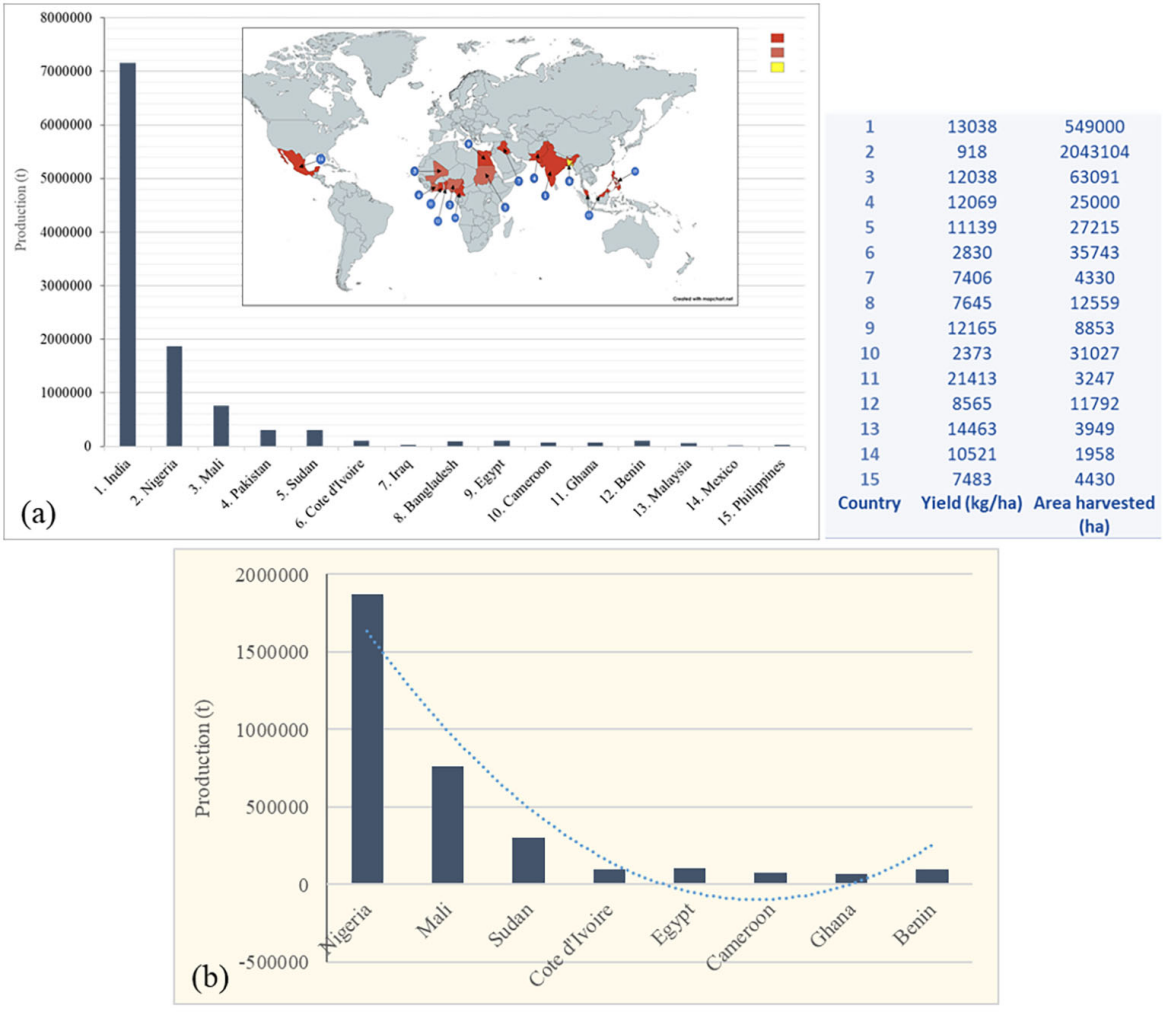
The top global and regional (Africa) okra production and yield statistics. **(a)** Top 15 country leaders in okra production globally. **(b)** Production based on agricultural land harvested in Sub-Saharan Africa where okra owes its origin (FAOSTAT, 2025).

Okra is considered one of the most marginalized but important vegetable crop of East African origin. Hypothetically, okra originated from one putative ancestor (*Abelmoschus ficulneus*) which is native to the Sahel region, including countries such as Niger and the northern part of Nigeria (Narkhede et al., 2014; Swamy, 2023). This crop is cultivated under subtropical and tropical climates, with most of its cultivation taking place in wide ranging soils under rain-fed and irrigated conditions (Yildiz et al., 2025). Okra is suitable for cultivation as both a garden crop and large-scale commercial farming. It is grown commercially in India, Nigeria and other parts of Africa, and America, especially Mexico as illustrated in **Figure 1**. According to the production statistics by Food and Agriculture Organization of the United Nations (FAOSTAT, 2025), India ranks first in world okra production with more than 7.1 million tons (70% of the total global production) per annum. As India remains the largest okra producer with peak production occurring between April and July. Patra et al. (2023) suggested that only a modest increase in yield on current area harvested could be achieved due to the lack and demand for high-yielding seed varieties. As a result, other major okra producing states (**Figure 1**) except India, focus on domestic supply instead of exports to international markets. This confirms the many existing agricultural deficits and challenges facing okra growers and breeding efforts in the already mentioned regions.

However, the global okra production market demonstrated consistent minimal increases, particularly, since 2020, while challenges such as the unavailability of uniform-sized seeds, low quality grains, poor yielding varieties and issues relating to storage, transportation and packaging constitute major problems in the okra industry. But, in Africa, as demonstrated in **Figure 1b**, okra production and yield also show marginal increases in the region that is proportional to the area harvested. There is limited statistics in many African countries such as South Africa, whose okra production has been concentrated in warmer regions like Limpopo Province. However, in South Africa, okra serves as one of the established crops grown with relatively low-maintenance, well-drained soil and plenty of sunlight with ideal temperatures between 22 to 30°C. Therefore, tackling these challenges, including the development of new high-yielding, stress resistant varieties, and optimization of agronomic practices remains a prerequisite for sustainable crop production (Patra et al., 2023). Furthermore, okra is well suited for cultivation in water resource-scarce areas, which implies that it reduces and improves water-use efficiency (WUE). With the global water crisis continuing to negatively impact human lives and food production, the WUE of crops such as okra is also very critical, and any crop with low water-use efficiencies will serve as one of the main concerns impeding sustainable crop production and productive agriculture.

## 3 Dealing with multi-stresses affecting okra cultivation, nutrition and health

Reducing the impact of multiple stresses that negatively affect okra production, nutritional content and potency, as well as the concentrations of phytochemicals comprising its health benefits requires the integration of modern agricultural biotechnology into okra breeding programmes. Currently, attempts to expand the okra gene pool has been achieved via interspecific cross breeding of which Yadav et al. (2025) indicated that it exhibit limitations of pre- and post- zygotic barriers. These barriers constitute restrictive mechanisms that prevent gene flow by blocking processes involved in the successful reproduction of fertile hybrids at different stages, often resulting in sterile offsprings (Wang and Filatov, 2023). Therefore, to address the frequency of sterility occurring in first filial generation (F1) hybrids onwards, integrated breeding, which involves novel tools and techniques for gene selection and incorporation into amenable hosts must be practiced. The adoption of this integrative approach will be able to provide the needed pace, precision and efficiency in accelerating breeding and delivery of newly improved okra varieties. This will also circumvent delays and long-breeding cycles involved in traditional hand emasculation and pollination that are presently responsible for the production of commercial hybrid seeds in okra and other crops (Suma et al., 2023; Wang and Filatov, 2023). Moreover, the adoption of this approach will improve the combating of biotic and abiotic stress factors that perpetuate the environment-related crop failures posing an immediate threat to agriculture, globally. The impact of these single or multiple stress effects in this crop and how they can be dealt with using modern technologies are discussed below.

### 3.1 Effect of biotic stress on okra

Okra requires longer, warm and humid growing conditions, because it is highly sensitive to frost and very low temperatures. However, many reports show that warmth and humidity create conditions that favors the growth and survival of many pathogens, including bacteria and fungi. Such conditions influence the pathogen’s ability to thrive and potentially cause diseases in plants. One such microbe is *Pseudomonas syringae*, a rod-shaped, Gram-negative bacterium that attacks crops by deploying a type III secretary system (T3SS) to inject effector proteins to suppress the plant’s immune system (Grenz et al., 2025). In okra, this bacterium causes bacterial leaf spot and blight, which result in reduced crop productivity and quality. Additionally, okra is also severely affected by a number of viral diseases caused by okra yellow vein mosaic virus (OYVMV), which is a monopartite *Begomovirus*. Monopartite begomoviruses are a type of plant virus that have a circular single-stranded DNA genome, and are known to cause various diseases in crops, including leaf curl and mosaic symptoms (Ouattara et al., 2022). This virus and other type of pests and pathogens summarized in **Table 1** have emerged as serious threats to many vegetable crops, especially okra in Africa and other parts of the world.

**Table 1:** A consideration of some of the major viruses, oomycete fungal diseases, nematode and arthropodous pests affecting okra production and yield (Abdi et al., 2019; Ouattara et al., 2022; Tayyab et al., 2024).

Classification	Common name/disease	Causative agent
Viruses	Okra yellow vein mosaic	*Begomovirus abelmoschusflavi*
	Radish leaf curl virus ((RALCV)	*Begomovirus* spp. *(RALCV)*
	Tomato spotted wilt virus (TSWV)	*Orthotospovirus tomatomaculae*
	Vermelhao disease	Luteoviridae, *Cotton anthocyanosis virus (CAV)*
	Okra yellow crinkle virus (OkYCV)	*Begomovirus gossypialabadense*
	Sida micrantha mosaic virus (SiMMV)	*Begomovirus sidamicranthae*
	Okra leaf curl virus (OLCV)	*Begomovirus- Bemisia tabaci*
Fungus and Fungi-like Oomycetes	Fruit rot	*Choanephora curcurbitarum*
	Stem canker	*Fusarium chlamydosporum sensulato*
	Fusarium wilt	*Fusarium oxysporum f. s. vasinfectum sensu lato*
	Alternaria pod spot	*Alternaria alternate (Fries) Keissler*
	Gray mold	*Botrytis cinerea*
	Powdery mildew	*Golovinomyces cichoracearum*
Arthropod and nematodes	Cotton aphid	*Aphid gossypii Glover*
	Whitefly	*Bemisia tabaci Gennadius*
	Cotton bollworm	*Helicoverpa armigera*
	Flea beetles	*Nisotra uniformis*
	Root-knot nematodes	*Meloidogyne* spp.
	Green plant bug	*Nezara viridula*
	Okra stem fly	*Melanagromyza hibisci*

### 3.2 Effect of abiotic stress on okra production

The abiotic stresses as environmental factors affecting crop production are not adequately combated for the immense scale and severity of disasters they cause in agriculture. In most cases, abiotic stresses cause sudden and devastating effects on crops, with widespread growth, yield and economic damage. Thus, understanding mechanisms of plant defense against these stressors and developing integrated combating strategies to cope with their impact supports sustainability efforts in this sector. Among the stresses, drought, higher temperatures, salinity and chilling stress form part of the crops’ cardinal abiotic stress constraints. Moreover, many research findings strongly suggest drought as a major stress and the most catastrophic challenge in agriculture (Wang et al., 2022a; Patra et al., 2023; Kopecka et al., 2023: Vermeulen et al., 2025). Similarly, Mkhabela et al. (2023) reported that drought stress influences plant performance by reducing gas exchange and altering chlorophyll fluorescence formation. They also observed that drought stress affected physiological processes that includes reduced stomatal conductance, transpiration rate, net carbon dioxide assimilation, and maximum quantum efficiency, effective quantum efficiency of PSII photochemistry, photochemical quenching and electron transport rate among the studied okra accessions. Moreover, the abovementioned physiological traits are useful in understanding breeding for drought-tolerance in okra since this stress remains a major constraint for breeders who focus on how the inefficient moisture supply causes reduction on crop production.

**Figure 2:**
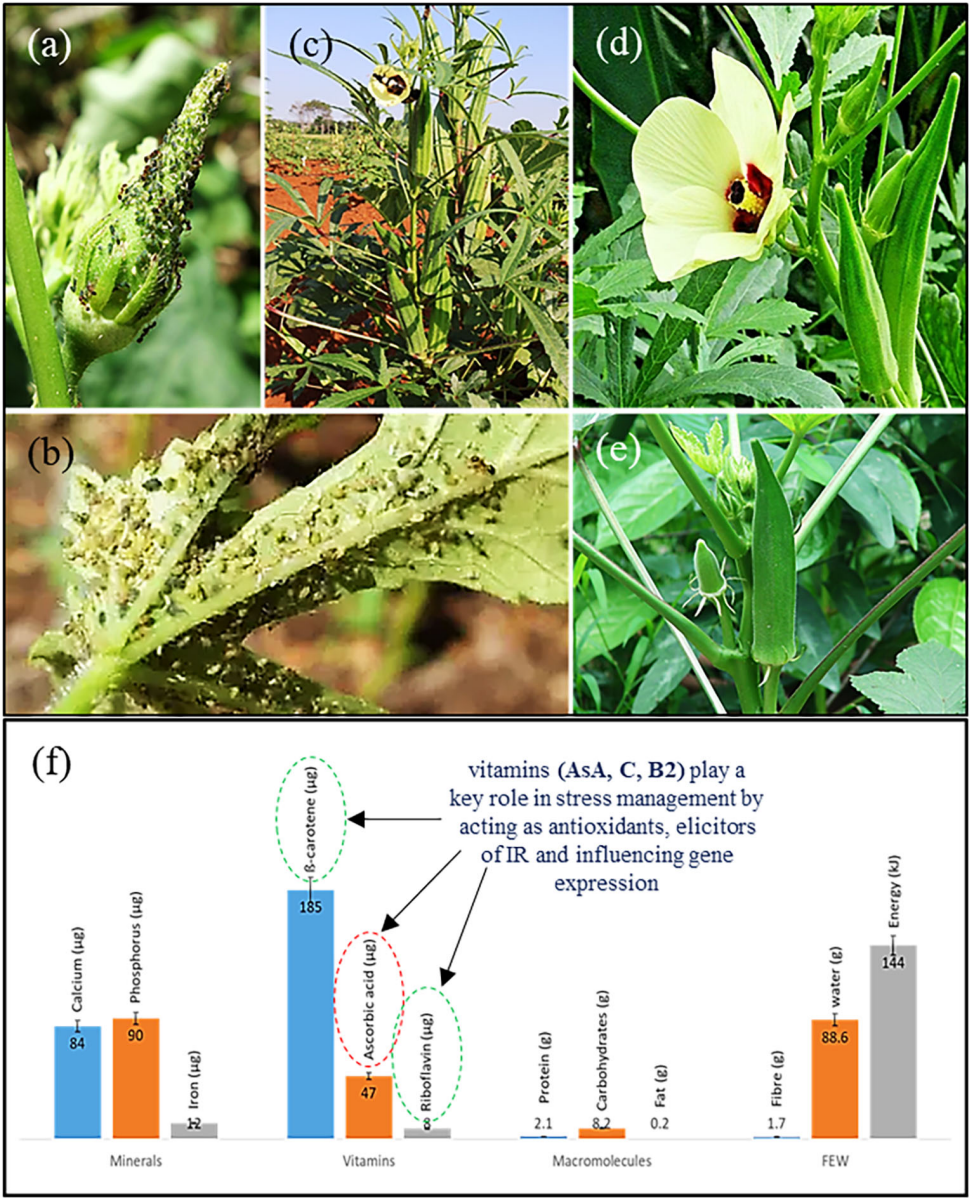
Okra plants (*A. esculentus*) showing immature pods and leaves attacked by **(a)***Formica* spp. (black ants), **(b)***Cicadellidae* (leafhoppers) and **(c)***Epicauta* sp. (blister beetles). Okra flower, floral bud and immature seed pods **(d, e)**, and raw nutrition value per 100 g of okra edible portion **(f)** (Romdhane et al., 2020; Swamy, 2023; Ounis et al., 2024; Tayyab et al., 2024).

Meteorologically, the drought phenomenon is classically estimated using a sophisticated system by an American National Weather Service Meteorologist Wayne Palmer, who developed the Palmer Drought Severity Index (PDSI or PDS Index). He created this index to provide a standardized way to measure drought based on precipitation and temperature over time (Venkatappa et al., 2021). Additionally, PDSI reflects the water balance by considering factors like potential evapotranspiration, soil moisture and runoff, making it particularly useful for monitoring agricultural drought. **Table 2** below emphasizes how low PDSI values lead to reduced crop yields, while excessive moisture (higher PDSI) negatively impact crops by accelerating pest attacks and disease spreads (**Table 1**; **Figure 2**). As reported by Venkatappa et al. (2021), the PDSI system also enables estimation of cropland and production damages that can be assessed and calculated as indicated in Equations 1, 2 shown below **Table 2** as per the affected area/region.

**Table 2:** Stress indicators and assessment of the impact of water stress on crop production using the Palmer Drought Severity Index analysis.

Stress indicator	PDS index	Effect
Near normal (drought)	0.44 to -0.49	Impact of stress noticeable and potentially disruptive.
Incipient drought	0.50 to -0.99	Initial stages of water scarcity with potential growth and yield losses.
Mild drought	-1.0 to -1.99	Tolerant, but leads to significant decreases in growth rates, yield and physiological/chemical changes.
Moderate drought	-2.0 to -2.99	Lead to significant decline in crop growth and productivity.
Severe drought	-3.0 to -3.99	Have devastating effects on crops.
Extreme drought	-4.0 to below	Lead to reduced yields and potential crop failure.
Extreme wetness/waterlogging	3.50 and above	Reduces oxygen availability, impacting plant growth and yield.
Severe wetness	2.50 to 3.49	Causes nutrient leaching and increasing the risk of diseases and pest infestation.
Mild to moderate wetness	1.00 to 2.49	Generally beneficial for crop growth as it promotes nutrient availability, enhances root development, and can improve overall yield.
Near normal (wetness)	-1.24 to 0.99	Generally beneficial for crops, but can negatively affect crops if it leads to waterlogging or excessive moisture.

Information compiled and presented on this Table was established from Venkatappa et al. (2021); Orimoloye (2022); Patra et al. (2023); Kopecka et al. (2023); Najafi et al. (2024) and Vermeulen et al. (2025).

(1) $$TCDA_{k}=\sum_{k=1}^{1}\sum_{i=1}^{6}CD_{ik}$$

(2) $$TCPl_{k}\;=\sum_{i=1}^{6}\left(CD_{ik}\times CR_{ik}\times CP_{k}\right)$$

In those equations, the total area of crop damage (TCDA_k_) in a specific region, designation (_k_) is quantified from the crop damage level CD_ik_, where i corresponds to PDSI level greater than 2.00 for flooding conditions or –2.00 signifying drought conditions in the region _k_. Furthermore, crop production losses or damage (TCPɭ_k_) by drought or flood in a region is estimated by applying the formula, where CP_k_ refers to crop production in the region (tons/year), and CR_ik_ for crop production reduction or proportion of crop damage, in terms of the crop damage level percentage (i) in the region. Like in many other crops, the Palmer’s index can be able to effectively estimate the long term drought impact on okra production with effects shown in terms of minus or plus relative values (**Table 2**).

Apart from drought, salinization as one of the cardinal stressors also severely impacts crop growth and productivity (Orimoloye, 2022). Saima et al. (2022) reported the effects of sodium chloride (NaCl) at 0, 25, 50 and 75 mM on Green Leaf, Rama Posa, Arka Anamika, Super Green, Okra Kashish and Nerali varieties of okra. The study pointed out that higher NaCl concentrations (>25 to 75 mM) resulted in poor to no okra growth, further reducing fresh and dry biomass of shoots and roots in all abovementioned varieties. While okra is considered a heat-tolerant crop, exceeding its 34 °C optimal growth temperature leads to reduced vegetative and physiological characteristics (Khalid et al., 2023). Comparing the physical and biochemical attributes of okra when subjected to chilling stress, Phornvillay et al. (2019) also reported inhibited growth, reduced yield and deteriorated fruit quality, in addition to chilling injuries sustained when okra pods were stored under low temperatures. The study further showed that, treating the pods with polyamines (Putrescine) reduced chilling injury by retarding the activity of poly phenol oxidase (PPO) and peroxidase (POD) by elevating phenolic content, DPPH (2,2-di-phenyl-1-picrylhydrazyl) radical scavenging activity, superoxide dismutase (SOD) and catalase (CAT) enzyme activity.

### 3.3 Multi-stress effects on nutritional value and yield quality in okra

As previously discussed, stress impact assessments assist in elucidating how crops and consumers are affected by the period of stress, particularly for species such as okra that serves as one of the most important vegetable crop supplementing the diet of many people in Asia and Sub-Saharan Africa. These regions are also considered climate change hotspots, characterized by abiotic stresses that affect crop production, singularly or more simultaneously, with secondary plant attacks by biotic stress factors. Therefore, both biotic and abiotic stresses, with consequential interaction with plant genes impacts on performance of this crops’ physiological system (Abdi et al., 2019). Generally, environmental stresses result in changes to ion balance, water potential, nutrients intake and photosynthetic efficiency, ultimately affecting cellular function and overall crop yield. Similarly, these changes were observed in okra where a chain of physiological perturbations, disturbed ion homeostasis and compromised photosynthetic efficiencies, initiating reductions in nutritional composition and quality of harvested produce (de Oliveira et al., 2025). Najafi et al. (2024) reported that levels of key nutrients in okra plants are influenced by salinity stress conditions, including antioxidants, minerals and vitamins, which then impact on human health and quality of food produced. However, research is limited in this regard, and there are conflicting findings in the classification of okra as a stress-sensitive or semi-tolerant crop due to the lack of specific stress tolerance threshold (Saima et al., 2022; Wang et al., 2024; de Oliveira Silva et al., 2025; Yang et al., 2025). Furthermore, analyzing the response of okra to drought stress, Wang et al. (2022c) highlighted that water deficit potentially reduces the availability of beneficial compounds, negatively impacting the various aspects of plant health and overall medicinal value. Since ancient times, infusions and decoctions of okra fruit pods have been used in folk medicine to promote human health (Romdhane et al., 2020). The edible plant parts, especially immature pods are nutritionally rich with ascorbic acid, carotenoids, thiamine, folic acid, riboflavin, oxalic acid, niacin, amino acids, minerals (potassium, calcium, phosphorus and magnesium) and edible dietary fiber (**Figure 2**). According to Younis et al. (2023) heat stress and drought significantly impacts on these chemical composition of okra fruits, especially leading to reductions in chlorophyll content and protein involved in their synthesis, while increasing proline and antioxidant activity. Meanwhile, insect pests like aphids, whiteflies and fruit borers can directly damage okra plants, causing reduced growth, deformed fruits and lower yields. Furthermore, diseases such as powdery mildew, *Fusarium* wilt and viral infections can cause nutrient deficiencies by interfering with the plant’s ability to absorb and utilize essential nutrients, reducing quality and nutritional content of the okra pods (Wang et al., 2022b; Ounis et al., 2024). It is also important to further highlight that these changes are always associated with reduced plant growth and yield. This requires prior understanding and knowledge of their causal agents for breeding purposes against these stresses.

## 4 Elucidating okra genome to combat multiple stress effects

Although, okra is perceived as a low-value and low market crop in some regions, this crop is quite popular in some countries due to the ease of cultivation, dependable yield and adaptability to varying environmental conditions. According to Swamy (2023), such reliable okra varieties that are cultivated in these regions are amphidiploids (2n = 130) containing a complete diploid set of chromosomes. This doubling of chromosome allows the plant to sexually reproduce and develop hybrids that exist as new independent species. Amphidiploids are typically allopolyploids, meaning they have multiple sets of chromosomes possessing growth and stress-resistant genes derived from different ancestral species (Suma et al., 2023). Okra varieties, as allopolyploids that evolved from diverged genomes are beneficial for adapting to changing environmental conditions and developing new crop varieties from their amphidiploid genetic base. This genetic advantage that okra and other *Abelmoschus* spp. contain offer breeders the opportunity to develop new cultivars with increased vigor, larger pods/plant organs, enhanced stress tolerance and broader genetic diversity. The abovementioned qualities are instrumental in the improvement of productivity, quality and overall yield for sustainable cultivation and commercialization of okra. In Africa, as illustrated in **Figure 1b** okra production and yield showed marginal increases in 2024 compared to 2021–2023 (data available at https://www.fao.org/faostat/en/#data/QCL).

Clearly, the increases are associated with the varieties’ genetic composition and potential. Presumably, the amphidiploid nature of varieties used earlier acted as a genetic bridge to transfer beneficial genes from wild okra relatives into cultivated okra. This may have improved yield-enhancing traits by facilitating the speciation of new varieties with improved growth and reproductive characteristics. Recent reports showed that polyploidisation may lead to intraspecific variations in traits associated with abiotic stress, and those include pronounced differences in root, shoot and numerous other quantitative stress responsive characteristics in plants (Martinez-Perez et al., 2003; Mokhtari et al., 2022). For instance, Islam et al. (2022) reported that polyploidy increased plant drought tolerance by effecting large cell sizes, altered stomatal density, improved hydraulic conductivity and stress-related gene expression and hormonal pathways in *Citrullus* spp. However, like in *Citrullus* spp. and any other plants, okra exhibit significant evolutionary ploidy changes at multiple levels in response to biotic and abiotic stresses. This include gene expression mediated changes, and metabolic accumulations (**Figure 2**) leading to the formation and prevention of reactive oxygen species (ROS) which induce oxidative damage (Liu et al., 2025). Muthaiah et al. (2024) also reported okra’s transcriptome sequencing and gene expression analysis that revealed both positive and negative regulation of genes in susceptible and stress resistant plants. The transcriptome sequencing and gene expression analysis involving secondary metabolites production are instrumental in providing crucial genomic resources and identifying genes linked to desirable traits like resistance to multi-stresses. In addition to enabling the development of improved cultivars through MAS and genomic selection. In elucidating the genome-based defence mechanism against these stresses, such as drought and YVMV, which caused substantial yield losses of up to 80–90% (Mubeen et al., 2021), it was found that okra’s extensive genomic deletions may have affected its biosynthesis of secondary metabolites required to trigger resilience against biotic stress factors and environmental adaptation. Similarly, this crop retained a substantial number of genes relating to metabolic content change across different okra varieties and developmental stages (Wang et al., 2023).

The response of okra to individual or multiple stress factors remains very complicated, and the molecular, as well as physiological mechanisms underlying this process are still ambiguous. These stresses result in carbohydrate metabolic pathways inhibitions, while influencing some of the secondary metabolic processes, either negatively or positively. Studies have also revealed that defence metabolites such as amino acids, carbohydrates (particularly glycosides and sugar esters), lipids, phenolics, terpenoids, alkaloids and glucosinolates constitute chemicals also found in okra and other plants. These metabolites are used as signaling molecules, osmolytes, osmoprotectants, and antioxidants, helping to neutralize harmful free radical scavenging activity occurring during the period of stress (Sandeep et al., 2022; Elkhalifa et al., 2021; Najafi et al., 2024). For example, in maize (Zea mays L.), key modifications of metabolite production through gene editing targeting specific genes like *Hahb4*, *CspA*, *CspB*, *NF-YB1*, *NF-YB2*, *TPS/TPP*, *TsVP*, *OsNACs*, *OsERF71*, *HVA1*, *ARGOS8* and *AtOSR7*, which have not been achieved in okra were reported (Rynjah et al., 2025). As an important source of highly nutritious chemicals, representing a good variation of proteins, vitamins, carbohydrates and minerals (shown in **Figure 2f**), okra’s rich nutritional composition, particularly vitamins such as ascorbic acid (AsA), riboflavin (vitamin B2) and ß-carotene (vitamin A) are crucial for stress responses, including various plant growth and development functions. Riboflavin for instance, plays a key role in stress management by supporting cellular energy production and acting as an antioxidant, potentially reducing stress associated oxidative damage. Against biotic stress, it acts as an activator of plant defense mechanism by triggering systematic resistance, priming plants for quicker and stronger defense response, and influencing gene expression related to stress management (Abdi et al., 2019; Sandeep et al., 2022).

Various reports on okra genetic improvement, including heterosis and combining abilities recommend streamlined breeding processes to enable efficient development of hybrids with enhanced metabolism to combat stressful conditions (Ranga et al., 2024; Liu et al., 2025). An earlier breeding study (Azami-Sardooei et al., 2010) demonstrated the effect of exogenous application of riboflavin on *Phaseolus vulgaris* plants, conferring resistance against *Botrytis cinerea*. Riboflavin applied at concentration of 10–100 μM reduced the number of spreading lesions by approximately 25% compared to the control. Ascorbic acid, known as vitamin C, also plays a crucial role by enhancing plant resistance to biotic stress. This vitamin acts as a potent antioxidant, scavenging ROS produced during pathogen attacks, and during the exposure of plants to abiotic stresses. Furthermore, AsA is involved in various signaling pathways, interacting with other antioxidants and phytohormones to activate defense responses against pathogens to promote the overall health of plants. However, Goggin et al. (2010) earlier argued that the abundance of vitamin C (L-ascorbic acid, ascorbate or AsA) in some instances influenced plant susceptibility to insect feeding. This study implied that plant pests such as ants (*Formica* spp.), blister beetles (*Epicauta* spp.) and leafhoppers (*Cicadellidae*) demonstrated attacking okra plants in **Figures 2a–c**, may do so while influenced by modifications of ascorbic acid contents. Therefore, to achieve the cultivation of healthier okra plants and pods as indicated in **Figures 2d, e**, showing lesser susceptibility to biotic and abiotic stress, integrating genomics with conventional breeding methodologies will enable direct genotype-phenotype analysis to improve growth and crop yield in okra. As this species (*A. esculentus*) is well known for its chromosome polymorphisms compared to its genetically unstable wild counterparts. The above differences are considered to be normal genetic variations, typically stable within individual species (with traits heritable in a Mendelian fashion) and could be utilized to develop elite new okra varieties with improved plant morphology, adaptability and evolutionary trajectory (Orimoloye, 2022; Swamy, 2023).

## 5 Effectively seizing the momentum of modern tools to reshape okra breeding

**Figure 3:**
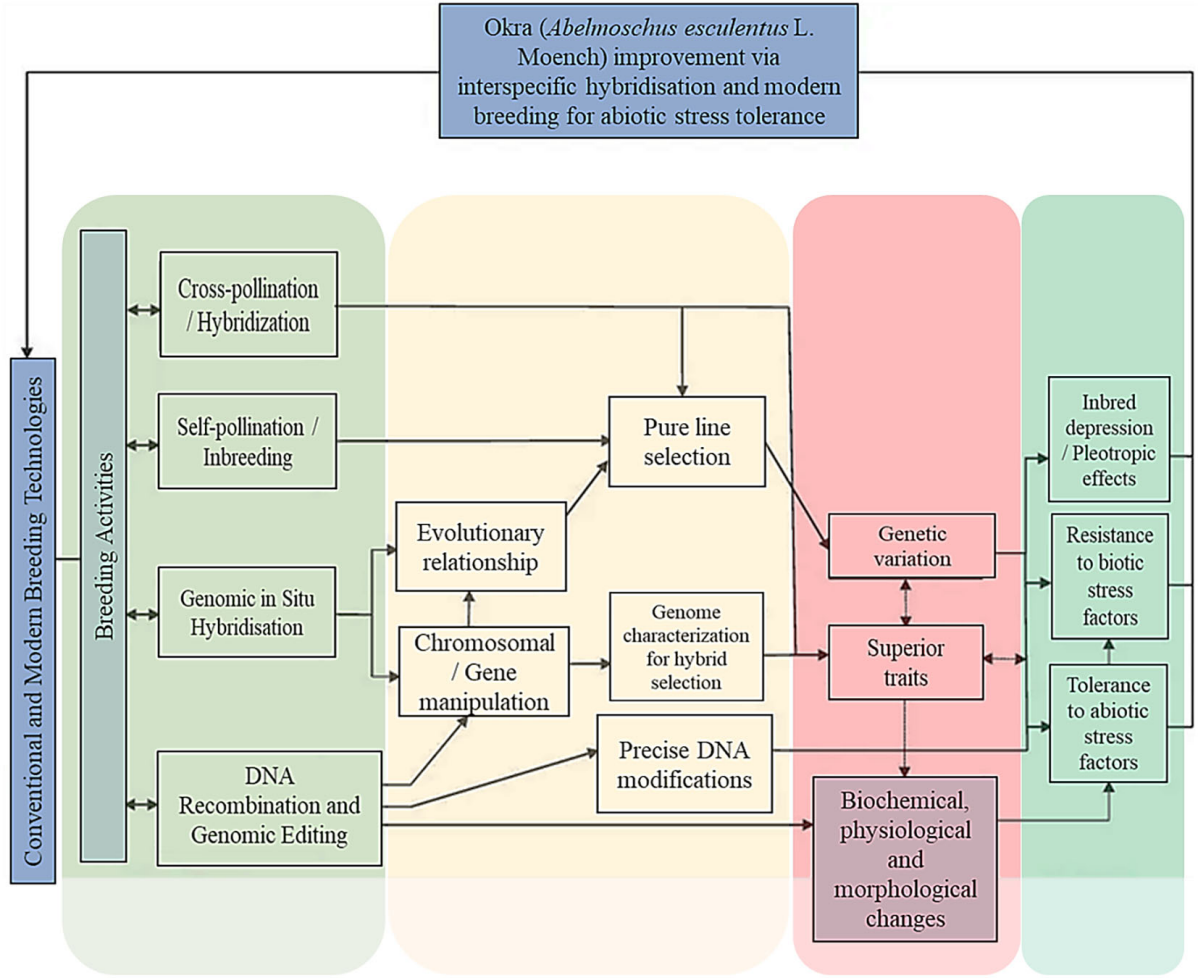
Potential application of genome based molecular breeding that harnesses advanced tools to enhance resilience to biotic and abiotic stress factors in okra (*A. esculentus* L. Moench.) for addressing agricultural challenges and increasing food security while dealing with obstacles like inefficient regeneration protocols, limited sequence data and difficulties in DNA isolation due to mucilage currently limiting widespread use compared to other crops.

As reported by Patil et al. (2022), Fernandes et al. (2022) and Martinez-Perez et al. (2003), chromosome polymorphism emanates from deletion and addition of one or few DNA sequences carrying the genetic information, which is tolerated in *A. esculentus*. Although, this provide better breeding opportunities by counteracting remarkable resilience to chromosome loss or gain seen in other crops excluding okra. These genetic characteristics have not yet been fully exploited in okra, like in many other grain crops for cultivar improvement. In wheat (*Triticum aestivum* L.) for example, certain chromosome deletions have been carried out and exploited in creating genetic stocks for breeding research. This has led to the development of hexaploid wheat (2n = 6x = 42) that currently remains the most common type of cultivated wheat known for its hard and soft kernels, and it is used in various food products like bread and pasta. Moreover, this wheat offers valuable genetic resources for improving stress tolerance in breeding programs (Mokhtari et al., 2022). Chromosome polymorphisms observed in okra, that is also autogamous in nature, also offers new avenues for breeders to restore genes lost during the evolutionary bottlenecks (Martinez-Perez et al., 2003). Okra also exhibits facultative allogamy, meaning it is susceptible to cross-pollination or controlled hybridization (**Figure 3**) of parents selected based on their better-combining characteristics (Swamy, 2023). Nonetheless, to date, it still not understood how adaptability, variation and possible selection for stress tolerance, especially in arid environment could take advantage of the evolution of *A. esculentus* from hybridization of *A. tuberculatus* and/or *A. ficulneus* in the past (**Figure 3**). *A. esculentus* has experienced genetic bottlenecks during domestication and then evolved with a narrow gene pool, perhaps losing a considerable portion of its original genetic diversity in the process. This may warrant exploration of interspecific crosses to create the exchange of genetic material and developing individuals with unique gene combinations. Interspecific hybridization is currently one of the most significant evolutionary phenomenon that can be used to potentially generate novel traits and increased genetic diversity, especially when coupled with modern tools such as molecular maker selection, genome editing and embryo rescue.

As mentioned earlier, these tools can help overcome pre- and post-fertilization barriers, enhance gene transfer and accelerate the breeding process in okra. It was also evidence in studies of other crops that such hybrids have significant genetic variation for biotic and abiotic stress resistance, in addition to the agronomic and desirable quantitative traits (Cao et al., 2023; Sun et al., 2025; Vishwakarma et al., 2025). The molecular marker, such as the single nucleotide polymorphisms (SNPs, pronounced Snips) are the most common type of single-base genetic changes that can be used in identifying genomic regions linked with important traits to enhance breeding accuracy and efficiency (Kumar et al., 2024). In okra, these are found in genes like *Ae59G004900* and *Ae44G005470* that are involved in regulating seed germination, particularly under salinity stress (Xu et al., 2024). This genetic variation provides a tool for molecular marker-assisted breeding to develop salt-tolerant okra varieties. Such few reports exist, amid various researchers including Bhatnagar (2025) reporting that MAS in okra breeding faces significant limitations due to the crop’s inherent biological complexities and resource constraints. The allopolyploid nature and large size of its genome make the biology of this crop complex, redundant, and with high chromosome number resulting in difficult genetic mapping which hinders the identification of specific genes and reliable molecular markers essential for effective MAS application. Furthermore, techniques like Clustered Regularly Interspaced Short Palindromic Repeats- associated protein 9 (CRISPR-Cas 9) also allow precise gene modification to accelerate breeding. Although, this technique has not been widely applied in okra, due to inefficient DNA modifications/delivery methods and off-target mutations as a result of genome complexity. It promises to address improvement constraints such as regeneration inefficiencies, limited sequence data, and difficulties in DNA isolation due to mucilage properties found in this crop (**Figure 3**). Nevertheless, this and other genomic tools can be explored and urgently prioritized to improve breeding, for purposes of combating a myriad of risks posed by biotic and abiotic stresses.

## 6 Future perspectives and conclusion

The advent of genomic tools currently used for molecular breeding have revolutionized the art and science of improving plants by manipulating their genetics to enhance desired traits such as yield, grain quality and stress resistance. Most crucially, the fact that the global population and adverse effects of climate change are increasing, necessitate increased food production and the need for high-yielding stress resilience cultivars. Genome engineering techniques are therefore, needed to guide the development and production of stress resistant plants, especially for vegetable crops such as okra that emergingly constitute a significant portion of the human diet in various parts of the world. Given the rising interest of consumers and industries into okra, it is thus essential to ensure their sustainability in the face of biotic and abiotic threats, while commercially producing cultivars with valuable traits such as enhanced nutrition and reduced antinutritional components post harvesting. Kushwaha et al. (2025) emphasized integration of emerging technologies such as protoplast-based CRISPR/Cas system with breeding to overcome agricultural challenges. For vegetable crops like okra (*Abelmoschus esculentus* L. Moench), these modern breeding technologies are not yet widely explored, especially for major biotic stress factors like bacterial blights, fungal attacks, viruses, weeds, insect pests (**Figure 2**) and abiotic stresses (drought, salinity, cold stress, heat stress and light stress, **Table 2**). Thus, these environmental constraints will continue to undermine conventional breeding efforts if innovative breeding approaches are not adopted. Modern genomic breeding is guaranteed to offer efficiency, precision, specificity, affordability, productivity and sustainability in the breeding of okra and other vegetable crops for multi-stress resistance, particularly better nutrition and sustainability in agriculture.

## References

[B1] Abdi O. Shirvani Z. Buchroithner M. F. (2019). Forest drought-induced diversity of Hyrcanian individual-tree mortality affected by meteorological and hydrological droughts by analyzing moderate resolution imaging spectroradiometer products and spatial autoregressive models over northeast Iran. Agric. For. Meteorol. 275, 265–276. doi: 10.1016/j.agrformet.2019.05.029

[B2] Anand A. Subramanian M. Kar D. (2023). Breeding techniques to dispense higher genetic gains. Front. Plant Sci. 13. doi: 10.3389/fpls.2022.1076094, PMID: 36743551 PMC9893280

[B3] Azami-Sardooei I. Franca S. C. De Vleesschauwer D. Hofte M. (2010). Riboflavin induces resistance against *Botrytis cinerea* in bean, but not in tomato by priming for a hydrogen peroxide-fueled resistance response. Physiol. Mol. Plant Pathol. 75, 23–29. doi: 10.1016/j.pmpp.2010.08.001

[B4] Basheer L. Ben-Simchon E. Cohen A. Shelef O. (2021). From traditional food to functional food? Evaluation of Malvaceae species as novel food crops. Agron 11, 1294. doi: 10.3390/agronomy11071294

[B5] Bhatnagar M. (2025). “ Potential pre-breeding projects in okra for PPP collaborations,” in Okra: Status, Challenges and Opportunities. Eds. Tikoo S. K. Angadi S. Tiwari A. Yadav R. K. Tomar B. S. Adeniyi A. H. ( Springer, Singapore). doi: 10.1007/978-981-97-9963-3_24

[B6] Cao Y. He L. Song F. Li C. Ji Q. Liu J. . (2023). Physiological and gene expression response of interspecific hybrids of *Fraxinus mandshurica* × *Fraxinus americana* to MJ or SNP under drought. Forest 14, 1277. doi: 10.3390/f14061277

[B7] Das U. Islam S. (2019). A review study on different plants in Malvaceae family and their medicinal uses. Am. J. Biomed. Sci. & Res. 3, 94–97. doi: 10.34297/AJBSR.2019.03.000641

[B8] de Oliveira Silva M. A. Souza K. B. da Silva Costa E. dos Santos Souza L. F. de Oliveira Silva M. Varjao L. F. B. . (2025). Performance of commercial okra genotypes at salinity levels. Discov. Plants 2, 25. doi: 10.1007/s44372-025-00110-w

[B9] Elkhalifa A. E. O. Alshammari E. Adnan M. Alcantara J. C. Awadelkareem A. M. Eltoum N. E. . (2021). Okra (*Abelmoschus Esculentus*) as a potential dietary medicine with nutraceutical importance for sustainable health applications. Molecules 26, 696. doi: 10.3390/molecules26030696, PMID: 33525745 PMC7865958

[B10] Fernandes F. M. de Queiroz M. V. da Silva L. L. Azevedo D. M. Q. Badel J. L. Alfenas A. C. (2022). Chromosomal polymorphism of the Ceratocystis fimbriata species complex in Brazil. Fungal Genet. Biol. 162, 103728. doi: 10.1016/j.fgb.2022.103728, PMID: 35932991

[B11] Food and Agriculture Organisation of the United Nations (FAO) Statistics (2025). Crops and livestock products. Available online at: https://www.fao.org/fapstat/en/data?/QCL (Accessed September 2, 2025).

[B12] Goggin F. L. Avila C. A. Lorence A. (2010). Vitamin C content in plants is modified by insects and influences susceptibility to herbivory. Bioessays 32, 777–790. doi: 10.1002/bies.200900187, PMID: 20665764

[B13] Grenz K. Chia K.-S. Turley E. K. Tyska A. S. Atkinson R. E. Reeves J. . (2025). A necrotizing toxin enables *Pseudomonas syringae* infection across evolutionarily divergent plants. Cell Host Microb. 33, 20–29. doi: 10.1016/j.chom.2024.11.014, PMID: 39706183

[B14] Iqbal Z. Iqbal M. S. Hashem A. Abd-Allah E. F. Ansari M. S. (2021). Plant defense responses to biotic stress and its interplay with fluctuating dark/light conditions. Front. Plant Sci. 12. doi: 10.3389/fpls.2021.631810, PMID: 33763093 PMC7982811

[B15] Islam M. Deepo D. M. Nasif S. O. Siddique A. B. Hassan O. Siddique A. B. . (2022). Cytogenetics and consequences of polyploidisation on different biotic-abiotic stress tolerance and the potential mechanisms involved. Plants 11, 2684. doi: 10.3390/plants11202684, PMID: 36297708 PMC9609754

[B16] Khalid U. B. Shah B. H. Ahmed I. Ali M. Ayyub S. Ahmad M. (2023). Temporal heat stress mitigation and physiological response in *Abelmoschus esculentus* L. by foliarly supplied salicylic acid. J. Pure Appl. Agric. 8, 61–69.

[B17] Kopecka R. Kameniarova M. Cerny M. Brzobohary B. Novak J. (2023). Abiotic stress in crop production. Int. J. Mol. Sci. 24, 6603. doi: 10.3390/ijms24076603, PMID: 37047573 PMC10095105

[B18] Kumar R. Das S. P. Choudhury B. U. Kumar A. Prakash R. N. Verma R. . (2024). Advances in genomic tools for plant breeding: Harnessing DNA molecular markers, genomic selection, and genome editing. Biol. Res. 57, 80. doi: 10.1186/s40659-024-00562-6, PMID: 39506826 PMC11542492

[B19] Kushwaha S. B. Nagesh C. R. Lele S. S. Viswanathan C. Prashat G. R. Goswami S. . (2025). CRISPR/Cas technology in vegetable crops for improving biotic, abiotic stress and quality traits: Challenges and opportunities. Scientia Hort 341, 113957. doi: 10.1016/j.scienta.2025.113957

[B20] Lamichhane S. Thapa S. (2022). Advances from conventional to modern plant breeding methodologies. Plant Breed Biotechnol. 10, 1–14. doi: 10.9787/PBB.2022.10.1.1

[B21] Liu W. Wang J. Zhu D. Yin X. Du G. Qin Y. . (2025). Jasmonic acid-mediated antioxidant defense confers chilling tolerance in okra (*Abelmoschus esculentus* L.). Plant 14, 1100. doi: 10.3390/plants14071100, PMID: 40219168 PMC11991441

[B22] Martinez-Perez E. Shaw P. Aragon-Alcaide L. Moore G. (2003). Chromosomes form into seven groups in hexaploid and tetraploid wheat as a prelude to meiosis. Plant J. 36, 21–29. doi: 10.1046/j.1365-313x.2003.01853.x, PMID: 12974808

[B23] Mkhabela S. S. Shimelis H. Gerrano A. S. Mashilo J. (2023). Drought tolerance assessment of okra (Abelmoschus esculentus [L.] Moench) accessions based on leaf gas exchange and chlorophyll fluorescence. Life 13, 682. doi: 10.3390/life13030682, PMID: 36983840 PMC10052028

[B24] Mokhtari N. Majidi M. M. Mirlohi A. (2022). Potentials of synthetic hexaploid wheats to improve drought tolerance. Sci. Rep. 12, 20482. doi: 10.1038/s41598-022-24678-5, PMID: 36443382 PMC9705419

[B25] Mubeen M. Iftikhar Y. Abbas A. Abbas M. Zafar-ul-Hye M. Sajid A. . (2021). Yellow vein mosaic disease in okra (*Abelmoschus esculentus* L.): An overview on causal agent, vector and management. Phyton-Int J. Exp. Bot. 90, 1573–1587. doi: 10.32604/phyton.2021.016664

[B26] Mustafa M. S. Rashid S. Gulnaz N. Saleen U. Noor G. Shakoor N. . (2021). A detailed review of conventional and modern breeding technologies and approaches of field crops. Asian J. Biotechnol. Genet. Engineer 4, 162–174. Available online at: https://doi.org/journalajbge.com/index.php/AJBGE/article/view/72 (Accessed October 11, 2025).

[B27] Muthaiah G. Mottaiyan P. Reddy M. R. Ravishankar K. V. (2024). Transcriptome sequencing and gene expression analysis reveals differential expression in response to YVMV infection in contrasting genotypes of okra. Scientia Hort 337, 113432. doi: 10.1016/j.scienta.2024.113432

[B28] Najafi R. Rezaei A. Mozafarian M. (2024). Physiological and biochemical responses of okra (*Abelmoschus esculentus*) under salinity stress in Iran. J. Agric. Food Res. 18, 101322. doi: 10.1016/j.jafr.2024.101322

[B29] Narkhede G. W. Deshmukh S. B. Shinde S. M. (2014). Wild relatives of okra (*Abelmoschus* spp.)- An overview. Eco. Environ. Conserv. 21, 301–306.

[B30] Orimoloye I. R. (2022). Agricultural drought and its potential impacts: Enabling decision-support for food security in vulnerable regions. Front. Sustain. Food Syst. 6. doi: 10.3389/fsufs.2022.838824

[B31] Ouattara A. Tiendsebeogo F. Becker N. Urbino C. Thebaud G. Hoareau M. . (2022). Synergy between an emerging monopartite *Begomovirus* and a DNA-B component. Sci. Rep. 12, 695. doi: 10.1038/s41598-021-03957-7, PMID: 35027584 PMC8758689

[B32] Ounis S. Turoczi G. Kiss J. (2024). Arthropod pests, nematodes, and microbial pathogens of okra (*Abelmoschus esculentus*) and their management: A Review. Agron 14, 2841. doi: 10.3390/agronomy14122841

[B33] Patil P. G. Jamma S N. M. Bohra A. Pokhare S. Dhinesh Babu K. Murkute A. A. . (2022). Chromosome-specific potential intron polymorphism markers for large-scale genotyping applications in pomegranate. Front. Plant Sci. 13. doi: 10.3389/fpls.2022.943959, PMID: 36110362 PMC9468638

[B34] Patra S. K. Paddar R. Pramanik S. Bandopadhyay P. Gaber A. Hossain A. (2023). Growth, yield, water productivity and economics of okra (*Abelmoschus esculentus* L.) in response to gravity drip irrigation under mulch and without mulch conditions. Scienta Hort 321, 112327. doi: 10.1016/j.scienta.2023.112327

[B35] Phornvillay S. Pongprasert N. Wongs-Aree C. Uthairatanakij A. Srilaong V. (2019). Exogenous putrescine treatment delays chilling injury in okra pod (*Abelmoschus esculentus*) stored at low storage temperature. Scientia Hortic. 256, 108550. doi: 10.1016/j.scienta.2019.108550

[B36] Prabhu K. R. Kumar A. Yumkhaiban R. S. Janeja H. S. Krishma B. Talekar N. (2023). A review on conventional and modern breeding approaches for developing climate resilient crop varieties. J. Appl. Nat. Sci. 15, 978–997. doi: 10.31018/jans.v15i3.4653

[B37] Ranga A. D. Vikram A. Kumar R. Dogra R. K. Sharma R. Sharma H. R. (2024). Exploitation of heterosis and combining ability potential for improvement in okra (*Abelmoschus esculentus* L.). Sci. Rep. 14, 24539. doi: 10.1038/s41598-024-75764-9, PMID: 39424932 PMC11489658

[B38] Romdhane M. H. Chahdoura H. Barros L. Dias M. I. Carvalho Gomes Corrêa R. Morales P. . (2020). Chemical composition, nutritional value, and biological evaluation of Tunisian okra pods (*Abelmoschus esculentus* L. Moench). Molecules 25, 4739. doi: 10.3390/molecules25204739, PMID: 33076530 PMC7587556

[B39] Rynjah D. Sandhanam K. Bhattacharjee B. Deka B. Newar A. Kalita T. . (2025). CRISPR/Cas9 gene editing systems for enhancing secondary metabolite biosynthesis via reproductive tissue modification. Discov. Plants 2, 245. doi: 10.1007/s44372-025-00334-w

[B40] Saima S. Ghaffar F. Yasin G. Nawaz M. Ahmad K. M. (2022). Effect of salt stress on the germination and early seedling growth in Okra (*Abelmoschus esculentus*). Sarhad J. Agric. 38, 388–397. doi: 10.17582/journal.sja/2022/38.2.388.397

[B41] Sandeep P. Priyamvada B. Tiwari M. Singh S. (2022). Effect of abiotic stresses on growth and metabolism of the plant and stress tolerance mechanism. Int. J. Sci. Adv. Res. Technol. 9, 1–7.

[B42] Suma A. John K. J. Bhat K. V. Latha M. Lakshmi C. J. Pitchaimuthu M. . (2023). Genetic enhancement of okra [*Abelmoschus esculentus* (L.) Moench.] germplasm through wide hybridisation. Front. Plant Sci. 14. doi: 10.3389/fpls.2023.1284070, PMID: 38023890 PMC10654990

[B43] Sun L. Ghouri F. Jin J. Zhong M. Huang W. Lu Z. . (2025). Interspecific hybridization enhanced tolerance to salinity and cadmium stress through modifying biochemical, physiological, and resistance gene levels, especially in polyploid rice: A sustainable way for stress-resilient rice. Rice 18, 19. doi: 10.1186/s12284-025-00776-6, PMID: 40119027 PMC11928717

[B44] Swamy K. R. M. (2023). Origin, distribution, taxonomy, botanical description, cytogenetics, genetic diversity and breeding of okra (*Abelmoschus esculentus* (L.) Moench.). Int. J. Dev. Res. 13, 26440. doi: 10.37118/ijdr.26440.03.2023

[B45] Tayyab U. B. Arif M. J. Khan M. D. Akhtar S. Abdullah M. J. Ali F. (2024). Tracking the feeding mechanism of sap-sucking insect pests through electropenetragraphy (EPG). J. Insc Behav. 37, 1–24. doi: 10.1007/s10905-024-09850-1

[B46] Venkatappa M. Sasaki N. Han P. Abe I. (2021). Impacts of droughts and floods on crop lands and crop production in southeast Asia- An application of Google Earth Engine. Sci. Total Environ. 795, 148829. doi: 10.1016/j.scietotenv.2021.148829, PMID: 34252779

[B47] Vermeulen S. Cools J. Passal S. (2025). Economic effects of drought on agriculture: Conceptual methods and stakeholders’ perceptions. Int. J. Dis. Risk Red 116, 105073. doi: 10.1016/j.ijdrr.2024.10503

[B48] Vishwakarma P. K. Vasugi C. Varalakshmi L. R. Shivashankara K. S. (2025). Screening of *Psidium* species and interspecific hybrid progenies for salinity stress tolerance. J. Plant Growth Regul. 44, 251–2366. doi: 10.1007/s00344-024-11551-0

[B49] Wang L. Filatov D. A. (2023). Mechanisms of prezygotic post-pollination reproductive barriers in plants. Front. Plant Sci. 14. doi: 10.3389/fpls.2023.1230278, PMID: 37476168 PMC10354421

[B50] Wang L. H. Gao M. K. Adnan R. Hassan M. U. Hassan M. A. Muhammad I. U. H. . (2024). Putting plant growth-promoting microbes in action effective way to mitigate salinity stress in plants: Review and future perspective. Appl. Ecol. Environ. Res. 22, 4481–4505. doi: 10.15666/aeer/2205_44814505

[B51] Wang F. Lai H. Li Y. Feng K. Zhang Z. Tian Q. . (2022c). Dynamic variation of meteorological drought and its relationships with agricultural drought across China. Agric. Water Manag 261, 107301. doi: 10.1016/j.agwat.2021.107301

[B52] Wang R. Li W. He Q. Zhang H. Wang M. Zheng X. . (2023). The genome of okra (*Abelmoschus esculentus*) provides insights into its genome evolution and high nutrient content. Hort Res. 10, uhad120. doi: 10.1093/hr/uhad120, PMID: 37554345 PMC10405168

[B53] Wang J. Shi D. Bai Y. Zhang T. Wu Y. Liu Z. . (2022b). Comprehensive proteomic and metabolomic analysis uncover the response of okra to drought stress. PeerJ 10, e14312. doi: 10.7717/peerj.14312, PMID: 36444379 PMC9700456

[B54] Wang Z. Yang Y. Zhang C. Guo H. Hou Y. (2022a). Historical and future Palmer drought severity index with improved hydrological modeling. J. Hydrol 610, 127941. doi: 10.1016/j.jhydrol.2022.127941

[B55] Xu G. Cheng Y. Wang X. Dai Z. Kang Z. Ye Z. . (2024). Identification of single nucleotide polymorphic loci and candidate genes for seed germination percentage in okra under salt and no-salt stresses by genome-wide association study. Plants 13, 588. doi: 10.3390/plants13050588, PMID: 38475435 PMC10934433

[B56] Yadav R. K. Vinay N. D. Badiger M. Saakre M. Tomar B. S. Choudhary H. . (2025). “ Okra breeding: history, key milestones and challenges: Indian perspective,” in Okra Status, Challenges and Opportunities. Eds. Tikoo S. K. Angadi S. Tiwari A. Yadav R. K. Tomar B. S. Adeniyi A. H. ( Springer, Singapore), 95–111. doi: 10.1007/978-981-97-9963-3_9

[B57] Yang X. He J. Xu L. Kong M. Huo Q. Song J. . (2025). Salt gradient-driven adaptation in okra: uncovering mechanisms of tolerance and growth regulation. Front. Plant Sci. 16. doi: 10.3389/fpls.2025.1648092, PMID: 40772056 PMC12327092

[B58] Yildiz M. Sirke S. T. Kocak M. Mancak I. Ozkaya A. A. Abak K. . (2025). Characterization of a diverse okra (*Abelmoschus esculentus* L. Moench) germplasm collection based on fruit quality traits. Plant 14, 565. doi: 10.3390/plants14040565, PMID: 40006824 PMC11859941

[B59] Younis M. Akram N. A. Latef A. A. M. A. Ashraf M. (2023). Appraisal of improvement in physiological and metabolic processes by exogenously applied natural and synthetic ascorbic acid in okra (*Abelmoschus esculentus* L.) fruit subjected to water deficit stress. Phyton-Int J. Exp. Bot. 92, 2761–2784. doi: 10.32604/phyton.2023.028801

